# The stabilizing role of the Sabbath in pre-monarchic Israel:a mathematical model

**DOI:** 10.1007/s10867-014-9373-9

**Published:** 2015-01-18

**Authors:** Joseph Livni, Lewi Stone

**Affiliations:** 1 Omega-n Aviation, Science & Art Inc., 6875 Norwalk # 806, Cote-Saint-Luc, QC H4W 3G2 Canada; 2 Bio-Mathematics Unit, Department of Zoology, Tel Aviv University, Tel Aviv, 69978 Israel; 3 School of Mathematical and Geospatial Sciences, RMIT University, Melbourne, Australia

**Keywords:** SIRS epidemic model, Periodic forcing, Social dynamics, Nonlinear dynamics, Sabbath, Cultural anthropology

## Abstract

The three monotheistic cultures have many common institutions and some of them germinated in pre-monarchic Israel. Reasonably, the essential institutions were in place at that starting point; this work explores the possibility that the Sabbath is one of these institutions. Our mathematical examination points to the potential cultural, civic, and social role of the weekly Sabbath, that is, the Sabbath institution, in controlling deviation from social norms. It begins with an analogy between spread of transgression (defined as lack of conformity with social norms) and of biological infection. Borrowing well-known mathematical methods, we derive solution sets of social equilibrium and study their social stability. The work shows how a weekly Sabbath could in theory enhance social resilience in comparison with a similar assembly with a more natural and longer period, say between New Moon and Full Moon. The examination reveals that an efficient Sabbath institution has the potential to ensure a stable organization and suppress occasional appearances of transgression from cultural norms and boundaries. The work suggests the existence of a sharp threshold governed by the “Basic Sabbath Number ש_0_”—a critical observance of the Sabbath, or large enough ש_0_, is required to ensure suppression of transgression. Subsequently, the model is used to explore an interesting question: how old is the Sabbath? The work is interdisciplinary, combining anthropological concepts with mathematical analysis and with archaeological parallels in regards to the findings.

**Electronic supplementary material:**

The online version of this article (doi:10.1007/s10867-014-9373-9) contains supplementary material, which is available to authorized users.

## Introduction

Especially in recent years, mathematical models have proven to be important tools for shedding light on complex aspects of human behavior, culture, and communications, and for investigating topical anthropological questions. Their applications cover a wide spectrum of interest comprising, for example, the beginning of the transmission of learned human skills and its relation to population density and mobility [1]; the study of language selection in bilingual societies [2]; the effects of Computer Age social networks [3]; the problems of detached human groups as such that in Tasmania (which separated from Australia at the end of the glacial epoch) [4]; intriguing analogies between culture propagation and molecular diffusion [5]; and the compliancy of humans when confronted with different rules of behavior [6–10]. In a similar vein, this article demonstrates how mathematical modeling approaches may help understand justice administration systems of societies and their social organization, with a specific focus on the institution of the Sabbath and its appearance in the early history of Israel. Our approach takes advantage of the analogy between the spread of epidemics in human populations and the propagation of transgression (which we define here as violation of social norms) in human society.

Interestingly, similar contagion models have been formulated to express the propagation of any human behavior that is acquired by learning from others [5]. Before entering into further details of the model, it is necessary to understand the historical background of the problems involved, and we thus first outline some relevant concepts concerning societies in early Israel.

There is general consensus that early Iron Age Israel was a transient society composed of an association of tribes and clans [11–13]. Historians refer to this early period as pre-monarchic Israel; a period when Israelites had no central government and, in times of crises, were loosely guided by ad hoc chieftains known as Judges [12]. The precise time range of the period is controversial; however, it is generally approximated as between the 14th and 10th centuries BCE. Close to the 10th century BCE, the tribal groups amalgamated to form either one or two monarchies. In anthropological terms, transition organizations from tribes to states are called chiefdoms, or middle-range societies [14, 15]. Some of these transient societies shared an egalitarian ethos consisting of the conviction that all the members of the society are equal (age and gender discrimination notwithstanding). The archaeological evidence convincingly indicates that an egalitarian ethos existed in pre-monarchic Israel [13]. All cultural descendants of ancient Israel represented by the three monotheistic religions have, to some extent, kept the belief that all human beings are created equal. Furthermore, a few of these descendant societies also shared practical socio-political life styles characterized by a peer relationship between members rooted in the persuasion of being equal signatories to a covenant. The term covenant signifies a binding code attributed to a divine source of law setting imperatives of lawful behavior in a society ruled by an assembly government. The code consists of norms such as prohibition of idolatry, prohibition of working on the Sabbath, sometimes prohibition of certain foods, and a multitude of other commandments regulating public life, justice administration, relations of a congregation with other congregations, and the behavior of individuals within their family and within their congregation.

These types of societies have been studied by scholars of political science and termed “covenantal societies” [16–19]. The view that these settlements on the hills of Samaria and Judea are the archetype of all covenantal societies is not new [20], yet for our purpose it is not necessary to entail that the covenant was specifically the one of the Old Testament (see Table 1).

**Table 1:** Examples of typical covenantal cultures

Name	Covenant	Office holders	Reference
Rabbinical Judaism	Torah;Torah-she-Baal-Peh	Zekenim; Parnasim; Vaad Hair; Maamad	Deut 20:6 [21]
Early Christians	Christian Bible; Decrees of Council	Presbyteros; episkopos	[22, 23]
Vaudois Sect	Christian Bible; Discipline Vaudoise; ordonnances ecclésiastiques	Regidors; Preires; (Presbyters, anciens); Barbas (pasteurs)	[24–26]
First Calvinists^a^	Christian Bible; Ecclesial Ordinances; Edicts of the Lieutenant (of Justice); Ordinances of Offices and Officers	Ruling elders; pastors, procurators	[27, 28]
Scottish Presbyterians	Christian Bible; Westminster Confession	Elders; Ministers	[29, 30]
English/ American Puritans^b^	Christian Bible; Puritan Confession	Elders; Ministers; Pastors	[31, 32]
Pilgrims	Christian Bible; Puritan Confession; Mayflower Compact	Elder; Pastor	[33, 34]

^a^All the other examples forbade working on the Sabbath; the First Calvinists specifically distinguished the Sabbath as a day of public worship

^b^With regard to the Puritans, it is worthwhile to quote clergyman Norton’s description of his New England frontier experience as: “holding forth a pregnant demonstration of the consistency of Civil-Government with a Congregational-Way” [31]. This is an astonishingly concise expression of the covenantal concept; the association of frontier society with a covenantal or egalitarian system is also observed by Elazar [16–20] and by Faust [13]

A fundamental difficulty of all sedentary egalitarian societies, including covenantal societies, is the capability to administer justice without creating institutions such as police and jail keepers that contradict egalitarian values. Different egalitarian cultures adopted different solutions. For example, the Mohawks adopted a system in which social deviation comes into being when the victim declares feud against his perpetrator. Diagonally opposed to the covenantal system where transgression, guilt, and consequential punishment play an important role in keeping its members on track, the Longhouse Justice System solves the problem by mediation between perpetrator and victim, through an arbitration that “*is not a process grounded upon fact*-*finding toward a determination of guilt and allocation of punishment*,” [35–37]. In Arab towns and villages, there is a similar system of offense, followed by a cycle of retributions ending with a negotiated “*sulkha*,” or settlement ceremony [38]. This tradition also appears to solve problems in a society with no police. In this paper, we make use of a mathematical modeling approach to help understand how egalitarian societies have persisted without the need of pervasive policing or the enforcement of justice systems.

A detailed study of egalitarian societies is beyond the scope of this article. However, Rousseau (pers. comm.; also see [15]) provided a few examples of the way such societies treat unruly behavior: some (Iban tribes), do nothing and their villages keep splitting and recombining—the villages are short-lived; others react to unruliness by constant negotiations (Kayan nation); in other tribes the chief increases his authority and the society starts its conversion to stratification (Trobriand tribes). That other societies adopted other solutions, and some adopted no solution, partially explains the lack of social stability of most egalitarian cultures. (Shortly, we will provide more formal definitions of stability and make use of the concept of mathematical stability.) However, some covenantal cultures did last for surprisingly long periods. At least three of the examples of Table 1 led an autonomous life for centuries as far as justice administration is concerned. Archaeological excavations in Israel [13] indicate that during the 300 years from early settlement to the formation of monarchy, the settlers had a classless organization, surprisingly paralleling the Biblical claim that “there was no king in Israel; every man did that which was right in his own eyes” [Judges 21:25]. No wonder this atypical kingless sedentary society captivated the scholars. For example, Hackett [12] remarks that Gideon’s refusal to become King is the right attitude for those times. In other words, regardless of the historicity of Gideon’s story, the very fact that it circulated testifies that the no-king organization was not a matter of random choice, but a result of a conscious opposition to a mortal king. We would like to examine the mechanism behind such a long quasi-stability.

By definition, in a covenantal society the members share the conviction of being bound to the covenant. This conviction empowers congregants to impose on each other obedience, compliance, and repentance. Consequently, gatherings of the congregation, such as participation in the weekly Sabbath, significantly increase these effects of corrective action. Our *hypothesis* is that the essential stabilizing mechanism involved the institution of the Sabbath. This is consistent with the observation that all the examples of Table 1 strictly observed the Sabbath. Our research (see Supplementary Information SI1) revealed that John Calvin formulated this same hypothesis more than four centuries ago. Note that all three monotheistic cultures guarantee the freedom not to work on Sabbath at least to some extent. In fact, in a covenantal society, abstaining from work is not a freedom but an obligation.

Our work borrows anthropological terminology for studying the observation of law in different societies. For example, transgression is characterized by [39] as “conduct that breaks rules or exceeds boundaries” and “is a key idea for sociologists, anthropologists, and cultural theorists, and .. a major feature of postmodern thought.” Similarly, we are required to refer to the concept of repentance. “Individuals who have been excluded in some manner from society as a result of violating its laws or norms are often required to repent … in order to be readmitted” [40]; see also [41]. In addition, righteous behavior is defined as “obedience to the values and mores of one’s society” [42]

While we use these terms freely, it is important to realize that our goal is not to show that repentance and/or Sabbath actually saves society; instead, we explain that repentance and Sabbath function as law enforcement mechanisms in the presence of unacceptable behaviors or transgressions of individuals in society. In this respect, we follow anthropologists who report what they observe and understand in the behavior of the society they explore, something that usually requires borrowing terms of the investigated societies. In this respect, these terms should not be considered religiously charged or given any ethical value.

The Old Testament claims that the Sabbath was observed in pre-monarchic Israel (Supplementary Information SI1). However, the Old Testament has not been dated and its testimony is not considered as evidence. Moreover, in spite of the other documents of Supplementary Information SI1, there is no consensus about the age of the Sabbath. As knowledge about that period is limited, this investigation opens an additional avenue to learn about it.

The hypothesis that a periodic assembly can substitute for law enforcement implies the feasibility of several versions of covenantal societies because many cultures had regular assemblies at moon phases. This work will verify the feasibility of steady-state and stability when the corrective effect of the Sabbath assembly is approximated by a periodic function. The analysis provides some new mathematical results regarding periodically forced systems of this nature. In addition, the model shows that a short period between assemblies significantly improves the chances to reduce the negative impact of transgressors in society. Based on this result, the article reasons that the phases of the moon were too far apart and consequently the odd but sufficiently short 7-day period for the Sabbath prevailed.

## Models of stratified societies

Social deviation is in equilibrium with society’s reaction against it. This equilibrium is stable if increments of the disruptive elements are countered by adequate corrective measures that constrain unlawfulness within tolerable limits. Most modern societies achieve this by a complex set of corrective actions that include, among others, law enforcement, religion, education, social security, and health care. These concepts may be investigated mathematically, as, for example, in the work of Zhao et al. [6] who describe a complex dynamical model that includes the mutual effect that poverty, crime, and law enforcement have on each other [6]. In a similar spirit, the models of Epstein [7] and Rossi [8] explore the interrelationships of drug addiction, drug pushers, law enforcement, and prisoners.

Equation (1) is the mathematical expression of the simplest model that illustrates the dynamics of social deviation in the most basic justice system. This “predator–prey” model is borrowed from mathematical biology, but it has been extended and lies at the basis for the key standard models for studying human societies [6–10]:

1 $$ \begin{array}{c}\hfill \frac{dx}{dt}= ax-bxy\hfill \\ {}\hfill \frac{dy}{dt}=cxy- dy\hfill \end{array} $$

Here, all the parameters *a*, *b*, *c*, and *d* are positive coefficients. In this model, *x*(*t*) represents the numbers of the criminal population at time *t*, while *y*(*t*) represents the size of the police force. The society reacts by incrementing or reducing the police size *y*, in a manner that keeps control of criminal deviants. In this case, according to the common underlying assumption of random population mixing, the police are able to come into contact and deal with criminals at a rate that is proportional to the product *xy*. In terms of the parameters, the average reduction rate of *x* due to one policeman catching criminals is *b*, while the growth ratio of the police per criminal is *c*. Note that in the absence of police the criminal population thrives and grows at per-capita rate *a*, while in the absence of criminals there is no need for a police force and it will decline at per-capita rate *d*.

The advantage of the mathematical formulation of the justice systems of (1) is that it leads to an unambiguous expression of the system’s conditions of equilibrium, stability, and dynamics in time. By definition, equilibrium is achieved when the derivatives of all variables with respect to time are zero. Inspection of Eq. (1) reveals that it has two equilibria; the trivial extinction equilibrium where everyone dies, both police and criminals (x* = y* = 0), and another positive equilibrium where police and criminals persist x* = *d*/*c* > 0 and y* = *a*/*b* > 0.

Stability of the equilibrium may be studied by determining if the system will return to equilibrium after a small perturbation. This is found by a mathematical procedure that requires examining the eigenvalues of the so-called Jacobian matrix of each of these equilibrium points [43]. It is enough that the real part of any eigenvalue is positive, for the system to be unstable; when all eigenvalues have real parts that are negative, stability is assured [43]. A zero eigenvalue represents neutral stability or a knifepoint case lying between stability and instability.

As the literature shows [43], the trivial extinction equilibrium is always unstable. This has the important implication that in this general type of stratified society, the police force and criminal subpopulations will never die out. Since the other (positive) equilibrium always has imaginary eigenvalues with no real parts, oscillatory dynamics between the police and criminals develop. As criminals rise in numbers, activity of the police force marches in step, increasing to contain the unlawfulness. The police are eventually able to reduce their size upon gaining control over the criminal elements. However, as police dwindle, a point is reached when crime pays yet again. The resulting “cat-and-mouse” game between police and criminals leads to continuous oscillatory waves of crime.

## A model of covenantal egalitarian societies

As our chief interest is the sustainability of typical egalitarian covenantal societies which have no police, the above model is completely unable to offer an explanation of how social deviation may be controlled in the absence of such law enforcers.

**Fig. 1:**
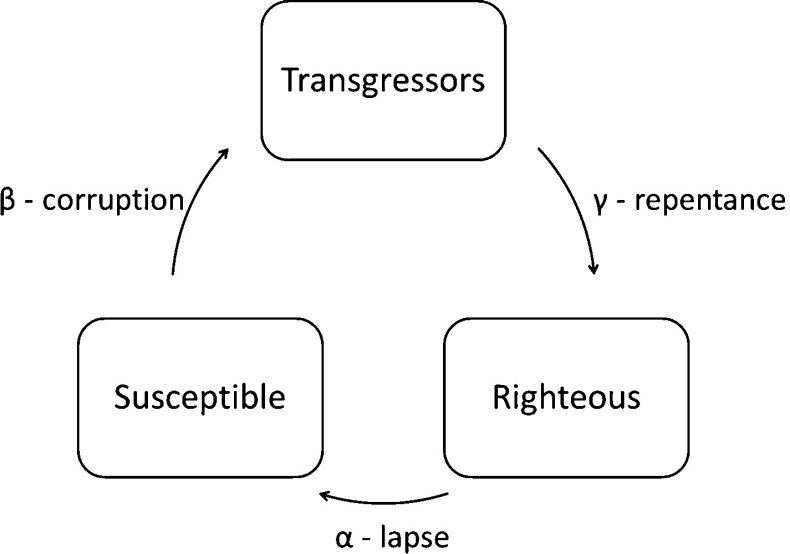
Compartmental diagram of the STRS model indicating the cycle of susceptible individuals who, upon corruption, join the transgressors. With repentance offered via the Sabbath assembly, transgressors (T) transform to righteous (R) individuals and in time may lapse to become susceptible (S) to corruption once again, thereby closing the STRS loop

For that reason, we propose to turn to Eq. (2), which is a variation of the well-known Kermack-McKendrick epidemiological model (Fig. 1). As mentioned earlier, the covenantal society is governed by an assembly; the assembly in general and each member of the congregation in particular are expected to obey a binding code referred to as a covenant (discussed above; see also SI-1c). In our case, an individual of the congregation regulated by a covenant can only belong to one of three classes: susceptibles (S) who may be corrupted, transgressors (T) who disobey, and righteous (R) who obey the covenant. The flow of individuals from class to class in this STRS model is illustrated in Fig. 1.

2 $$ \begin{array}{l}\frac{dS}{dT}=\alpha R-\beta ST/N\\ {}\frac{dT}{dt}=\beta ST/N-\gamma T\\ {}\frac{dR}{dt}=-\alpha R+\gamma T\end{array} $$

In this model, and in accord with the compartmental diagram of Fig. 1, susceptible individuals are corrupted by interaction with transgressors and join the transgressor class at a rate proportional to the product *ST*. After a period of time, followed by repentance, transgressors are able to become righteous and this occurs at a rate *γ* *T*. This expression represents an actual measurable proportion of *T* converting to *R*. Finally, righteous individuals may be tempted to lapse from their pious path to close the STRS loop and once again become susceptible to corruption at a rate *αR*.

The number of righteous individuals *R* quantifies the component of the population that cannot be swayed by transgressors to contravene the covenant. Here we suppose that any individual who determinedly obeys the covenant is righteous. His/her conviction may be moral, spiritual, religious, or simply based on some rationale. Similarly, repentance is stopping disobedience of the covenant and is controlled by the parameter *γ*. Since adding the three equations of the model gives $\frac{dS}{dt}+\frac{dT}{dt}+\frac{dR}{dt}=0$, the total population size *S* + *T* + *R* = *N* must be a constant, and *N* represents the size of the population of the covenantal organization. The time units of the model are taken to be weeks.

The proposed model needs to be consistent with our hypothesis that the repentance rate *γ* in a covenantal society is strongly connected to the Sabbath assembly. Before we examine an explicit periodic repentance function, we begin with a helpful approximation. We suppose that a Sabbath having period *k weeks*, i.e., appearing every *k weeks*, would ideally be able to ensure that the lifetime of a typical transgressor is k weeks on average. For the above model, it is not hard to show that transgressors remain in the T class on average for a period of time-length 1/*γ* (discussed further below). Thus in this first simplified model, a *weekly* Sabbath would require the life-time of a transgressor to be 1/*γ* = 1 week, although repentance could occur on any day of the week with equal chance.

Our following the more complex forced model attempts to synchronize the population dynamics by introducing a “clock” so that the transgressors are encouraged to repent specifically on the Sabbath. To achieve this, we deviate from the classical constant *γ* to a periodic one. While models with time-dependent transmission functions *β*(*t*) have been investigated in the past [44], as far as we know this is the first time an SIRS model is used with time-dependent *γ*(*t*).

### Model parameters

#### The transgression rate β

In an epidemic model, *β* represents the transmission of infection from an infected (I) individual to a susceptible (S) one at rate *βSI*. By analogy, we also adopted *βST*/*N* to model the propagation of social deviation and here the value of *β* measures the contagiousness of disobedience.

#### The lapse coefficient α

The parameter *α* is an idealized linear coefficient of the lapse loop (see Fig. 1). It expresses the rate at which righteous individuals become susceptible to transgression. Its epidemiological analogue expresses the rate of loss of immunity after infection and recovery from a disease. One can distinguish three social factors controlling the lapse rate:

1. Poverty increases *α* because it increases the rate of R → S conversions. This trend has been known from early covenantal existence [Deuteronomy 15:11] to the beginning of covenantalism in the New World [31]
2. Openness to foreign cultures increases *α* (this tendency also was known to early covenantal societies [Exodus 23:13]). This factor has ups and downs along history with decades of growth followed by decades of decline.
3. Temptation is a general term that comprises everything else; resistance to temptation is required with the transfer from nomadic pastoralist existence to sedentary life. This is because egalitarianism is more natural among pastoralists as observed by Schneider [45] and is explained by the fact that sedentary life allows for surpluses to be stored for worse times to come. Consequently it was suggested that a sedentary way of life lures some to accumulate surpluses on the account of others.

#### The repentance rate *γ*

In the epidemiological analogy, this term symbolizes the rate of recovery of infected people. In our social analogy, this becomes the repentance rate that converts T → R. As already discussed, we are able to choose the lifetime of a typical transgressor (the time in which a person is retained in the T-class) as 1/*γ* =1 week, to correspond to the 7-day Sabbath. But the rate may be changed to explore the effects of different Sabbath periods.

#### The Basic Sabbath number ש_0_ = *γ*/*βand cohesion factorγ*/*α*

Our investigation shows that these parameter ratios are sometimes more meaningful than the values of each separate parameter. In particular, the Basic Sabbath Number ש_0_ = *γ*/*β* (pronounced shin zero) is the reciprocal of the *basic reproduction number* (*β*/*γ*) in epidemiology. We might think of *β*/*γ* as the number of people a typical transgressor could corrupt in a totally susceptible naive population. In our application, its reciprocal, the Basic Sabbath Number (*γ*/*β*), indicates the resistance of a susceptible to be corrupted by a typical transgressor. Our results indicate that the Sabbath congregation holds back transgressors from seeding evil (lowers *β*) and increases repentance and righteousness (*γ*). Both of these healthy processes act to increase the Basic Sabbath Number ש_0_.

We will refer to the ratio *γ*/*α* as the *cohesion factor*, because the path to righteousness (*γ*) expresses cohesion while the lapse rate *α* acts to corrode the cohesion, e.g., under the influence of foreign cultures. The larger is the lapse rate, the smaller is the cohesion, and the higher is the rate that righteous individuals are transferred to the susceptible class.

### Estimating *β* for heroin addiction

An intriguing epidemiological modeling study of heroin addiction found that a population beginning with a single addicted person and 50 susceptibles ended up to be a combination of 25 affected and 25 susceptibles in 2.27 weeks to 5.3 weeks. Mackintosh and Stewart’s [46] simulation showed that this rate of addiction spread is equivalent to a 2% probability that an encounter of an affected person with a susceptible person results in the conversion of the susceptible into affected. In other words, on average an addicted person needs 50 encounters with susceptible individuals in order for one of these individuals to become addicted as well. We replicated these results in a simulation by inserting *N* = 50; *T*_0_ = 1; *S*_0_ = 50; *β* = 1.46, *α* = 0, *γ* = 0 in Eq. (2).

The simulation shown in Supplementary Information SI2 also indicates that the total susceptible population will become affected in approximately 5 weeks; this is consistent with data collected by Mackintosh and Stewart [46], indicating that 20–50 susceptibles become heroin addicts in a matter of weeks. Consequently, as a starting point, we adopted a simulation contagion rate for small congregations, which is approximately *β* = 1.5.

## Model analysis

### Equilibrium and stability of a covenantal society

Given the population size N is constant, it is possible to reduce the covenantal model to two differential equations with two variables (*S* and *T*) by eliminating the variable R, which can always be written as *R* = *N*-*S*-*T*. We arrive at the equations:

3 $$ \begin{array}{l}\frac{dS}{dt}=\alpha \left(N-S-T\right)-\beta ST/N\\ {}\frac{dT}{dt}=\beta ST/N-\gamma T\end{array} $$

The point of equilibrium requires that all variables have zero growth rates. This yields two equilibria whose stability is intimately controlled by the Basic Sabbath Number ש_*0*_ = *γ*/*β*

E1: The transgressor-free equilibrium T* = 0. By inspection of Eq. (3), one notes that a transgressor-free equilibrium exists in which:

4 $$ {\mathrm{S}}^{*}=\mathrm{N};\kern0.5em {\mathrm{T}}^{*}=0;\kern0.30em {\mathrm{R}}^{*} = 0 $$

In other words, the covenantal society is stationary with no transgressors, no righteous, and everybody susceptible. How long can a society stay at this stationary point? The answer is given by investigating the local stability at this point of equilibrium.

**Fig. 2:**
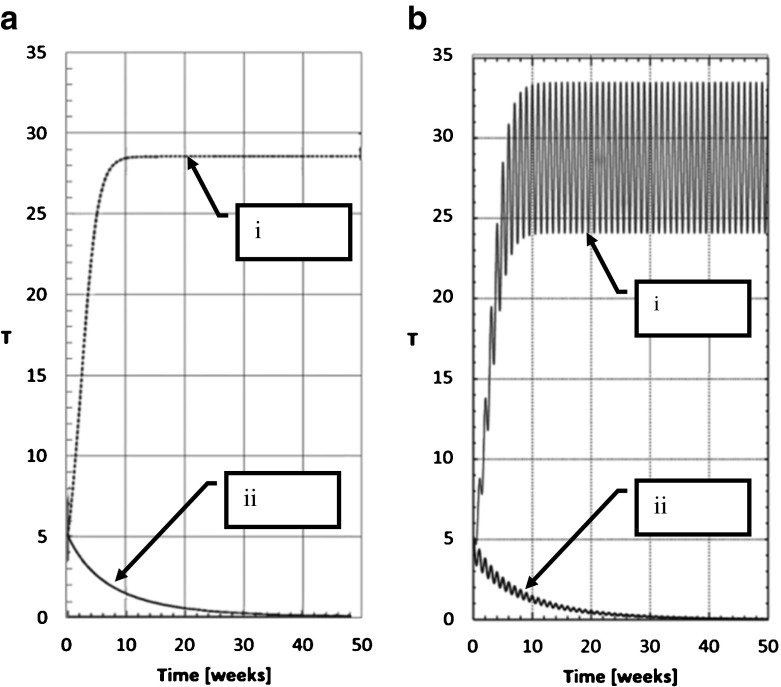
**a** Simulation results of a transgression wave contained by the repentance imposed by the pressure of the Sabbath assemblies in a congregation of *N* = 100 individuals. The line *i* represents ש_0_ < 1; transgressors rise fast in an epidemic, and finally stabilize to an endemic state. The line *ii* represents ש_0_ > 1; transgressors rapidly fall to zero. Parameters: cohesion factor =0.4; T_0_ = 5; ש_0_ = .6 (i); ש_0_ = 1.1 (ii). **b** Same as (**a**) but with periodical Sabbath *γ*(*t*) = 1 + sin(2*πt*). Note that the response is similar to the responses of (**a**) except for the occurrence of ripples on the STRS trajectories; this illustrates that for practical purposes the threshold results of the unforced model are representative. Parameters: cohesion factor =0.4; T_0_ = 5; ש_0_ = 0.6, (i); ש_0_ = 1.1, (ii)

The transgressor-free equilibrium is stable if both (real) eigenvalues are negative, which occurs if ש_0_ = *γ*/*β* > 1, (see Supplementary Information SI2). In this case, the number of transgressors will tend to decline until the transgression-free stationary point is reached, as shown in Fig. 2a (*i*). Note that when sitting at this equilibrium, all transgressors have been cleared from society, and the population stabilizes or locks down to a state totally free of transgression. In addition, in this regime, all the righteous eventually lose their status and become susceptible. Their memories of transgression and repentance, and thus their righteousness, erode with time. (Nevertheless, we shortly show how a direct route to righteousness can generate a transgressor-free community that includes a continuously maintained righteous subpopulation.)

Going further, if ש_0_ falls below unity then the equilibrium is unstable, and once the society is disturbed it will not return to its transgressor-free state, as shown in Fig. 2a (*ii*). This corresponds to an epidemic of transgressors in the original interpretation of Eq. (2).

E2: The equilibrium of endemic transgression: T* ≥0. The second endemic equilibrium point of Eq. (2) is easily found to be:

5 $$ \begin{array}{ccc}\hfill {\frac{S}{N}}^{*}=\frac{\gamma }{\beta };\hfill & \hfill \frac{T^{*}}{N}=\frac{1-\frac{\gamma }{\beta }}{1+\frac{\gamma }{\alpha }};\hfill & \hfill \frac{R^{*}}{N}=\frac{1-\frac{\gamma }{\beta }}{1+\frac{\alpha }{\gamma }}\hfill \end{array} $$

The equilibrium is only reasonable if ש_0_ = *γ*/*β* ≤1, ensuring that the transgressor population *T* ^*^ is non-negative. Note the interesting interpretation that if *γ*/*β* ≤1, then the proportion of susceptible congregants is exactly equal to the Basic Sabbath Number.

Similarly, the transgressors are proportional to (1-*S*/*N*) or to the proportion of non-susceptible. The transformation of the non-susceptible congregants into transgressors and righteous depends only on the *cohesion factor γ*/*α* (see Section 3). In a congregation with a cohesion factor *γ*/*α* > 1, the majority of non-susceptibles are righteous, otherwise the righteous are the minority. If the cohesion factor is one, then the non-susceptible split 50–50.

Consider now the stability at this stationary point of endemic transgression. Supplementary Information SI3 shows that if ש_0_ = *γ*/*β* < 1, both eigenvalues of the Jacobian always have negative real parts and the endemic transgression equilibrium is locally stable. This is reached either asymptotically or by damped oscillations.

**Fig. 3:**
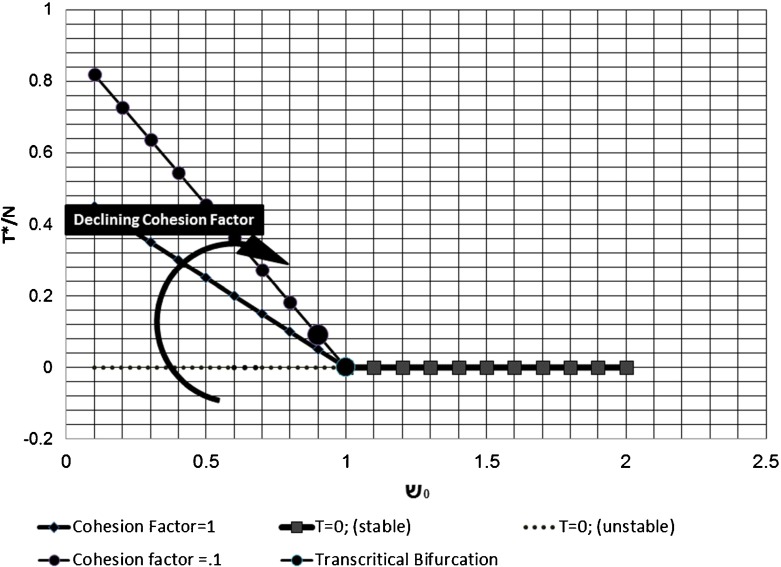
The transgressor equilibrium T* of the STRS model is plotted as a function of the Basic Sabbath Number ש_0_

As shown in Fig. 3, when ש_0_ > 1 the transgressor free equilibrium (T* = 0) is stable, while for ש_0_ < 1 the endemic transgression equilibrium (T* > 0) is stable. There is a transcritical bifurcation at ש_0_ = 1 where there is a change of stabilities between the equilibria. The figure indicates that a declining cohesion factor rotates the endemic transgression equilibrium line clockwise; at a zero cohesion factor the endemic transgression equilibrium coincides with the transcritical bifurcation point. A growing cohesion factor rotates the endemic transgression equilibrium counter-clockwise. As the cohesion factor approaches infinity the endemic transgression becomes almost transgression-free.

### The Sabbath threshold ש_0_

In summary, the STRS model of the covenantal society (3) has two equilibria governed by the Basic Sabbath Number ש_0_ = *γ*/*β* as portrayed in Fig. 3. When this number is unity, the model has a transcritical bifurcation [47]. There is an exchange of stability between these equilibria at the bifurcation so that for large Basic Sabbath Numbers (ש_0_ > 1) the covenantal society is transgression-free and stable. The model then predicts a congregation of 100% susceptible members with no transgressors.

When the Basic Sabbath Number falls below the Sabbath threshold point (ש_0_ < 1), the transgression-free equilibrium becomes unstable, and transgressors are able to proliferate until the congregation reaches a new equilibrium and the non-susceptible population will split according to the cohesion factor (see Eq. (5) and Fig. 3). The number of transgressors at equilibrium reduces as the cohesion factor increases. On this side of the bifurcation, the endemic transgression equilibrium will be stable with a constant component of corrupting transgressors.

Figure 2a shows simulation results of (3) comparing the responses (*S*, *T*) of a vigorous society with large Sabbath number (ש_0_ > 1) with the responses of a congregation that slipped into the endemic transgression domain ש_0_ < 1. The appearance of a small number of transgressors (*T*/*N* = 5%) is easily overcome by a quick return to a transgression-free equilibrium in the healthy society while the same small number grows to a high endemic transgression rate when ש_0_ moves above the threshold. In this way, the Sabbath threshold is the key determinant of the stability of the system. This section shows that as far as the mathematics is concerned, the model can function with a constant repentance mechanism. However, the covenantal reality offers no permanent corrective institution and our hypothesis is that the correction is related to the weekly assembly. Consequently, we now proceed to postulate a periodic Sabbath institution in the covenantal societies of Table 1.

## Effects of an explicit periodic Sabbath

Public life in a covenantal system flows to the beat of the weekly Sabbath pulsation. The working week in these societies is summed up by, and parallels, the Biblical quotation: “Six days you shall labor, and do all your work; but the seventh day is a Sabbath …” [Exodus 20:18]. When modeling a weekly Sabbath, it is reasonable to represent the repentance rate *γ* as a periodic function with the fundamental period of 1 week

6 $$ \gamma (t)=\overline{\gamma}+{\displaystyle {\sum}_{n=1}^{\infty }{\gamma}_n{e}^{ni\omega t}} $$

where $\overline{\gamma}$ symbolizes the time average or mean value of *γ*, *γ*_n_ symbolizes the amplitude of its nth harmonic and ω = 2π. Typically, one might take *γ*(*t*) = 1 + sin(2*πt*) as a very simple approximation whereby the forcing is sinusoidal with ω chosen to have period of 1 week, and the forcing amplitude given by δ. Of course, ω can be chosen to have any period we choose to investigate, and is not necessarily restricted to have a weekly period. Equation (3) becomes:

7 $$ \begin{array}{l}\frac{dS}{dt}=\alpha \left(N-S-T\right)-\beta ST/N\\ {}\frac{dT}{dt}=\beta ST/N-\gamma (t)T\end{array} $$

Supplementary Information SI3 illustrates that the interpretation of $1/\overset{\_}{\gamma }$is again the average time an individual remains a transgressor before he/she is able to repent sufficiently to move to the righteous class. For the simpler model Eq. (3), where *γ* is a constant, the transgressor-free society exists when ש_0_ ≥ 1 and below that value the society will settle at a stable T*/*N*. However, our analysis has not yet explicitly taken into account the periodicity of the Sabbath and we cannot be sure how this might affect the results so far. Could this periodic forcing jeopardize the stability condition entailed by the Sabbath threshold? In Supplementary Information SI3, we show that the model of (Eq. 7) has the same threshold as the unforced model:

8 $$ \begin{array}{l}\frac{dS}{dt}=\alpha \left(N-S-T\right)-\beta ST/N\\ {}\frac{dT}{dt}=\beta ST/N-\overline{\gamma}T\end{array} $$

We refer to Eq. (7) as *the forced model* and Eq. (8) as *the unforced model*. The Appendix shows that under an explicit periodic Sabbath the stability condition of the transgressor-free steady state is unchanged, namely ש_0_ $=\overset{\_}{\gamma }/\beta >1$, for both forced and unforced models. In addition, Supplementary Information SI3 shows that for all practical purposes the average steady state of the *S* and *T* responses to a periodic *γ*(*t*) are the same as the responses to a constant $\gamma =\overset{\_}{\gamma }$.

Figure 2b illustrates simulation results of the two congregations with *γ*(*t*) = 1 + sin(2*πt*) as compared to Fig. 2a. The simulations use a periodical time variant repentance rate and have the strongest possible amplitude of Sabbath forcing (given the constraint that always *γ*(*t*) ≥ 0). The results confirm that the average of the steady-state responses of the pulsating *γ* is practically the same as the steady state when a constant *γ* is used (Fig. 2a), and the threshold is unchanged, as detailed further in Supplementary Information SI3.

In summary, for both the simpler unforced model and the more realistic forced model, the same generic threshold is retained and similarly the same steady state values are found. The periodic forcing has little effect on these key characteristics. It does, however, offer a means of achieving a transgressor-free state through weekly Sabbath assemblies rather than corrective activities that are operating continuously every second of the day.

## Direct route to righteousness

In the above analysis, it was assumed that righteousness could only be achieved as an outcome of transgression. That is, a susceptible individual transgressed and through the aid of Sabbath repentance achieved righteousness. But another plausible route is through the ability of Sabbath to harden susceptible individuals to a degree that they can resist temptation of transgression, and move directly to the righteous class without ever transgressing. This direct route to righteousness is rather similar to a vaccination process whereby the Sabbath immunizes individuals from transgression.

We can model this as a weekly Sabbath pulse in which the proportion *p* of susceptible individuals move to the R compartment.

9 $$ \begin{array}{l}\frac{dS}{dt}=\alpha R-\beta ST/N-p{\displaystyle \sum_{n=0}^{\infty }S\left(n{T}^{-}\right)}\delta \left(t-n\tau \right)\\ {}\frac{dT}{dt}=\beta ST/N-\gamma T\end{array} $$

Here, τ is the time interval between Sabbath to Sabbath, which in usual practice we know to be 1 week. Pulses are applied via the Dirac delta-function and appear as impulses at the discrete times *t* = *n* *τ* (*n* = 0, 1, 2, …) and the moment immediately before the nth pulse is notated here as *t* = *nT* ^−^ The train of pulses generated by the delta function creates jump discontinuities in the variable S(t), which suddenly decreases by the proportion *p* whenever *t* = *n* *τ*.

**Fig. 4:**
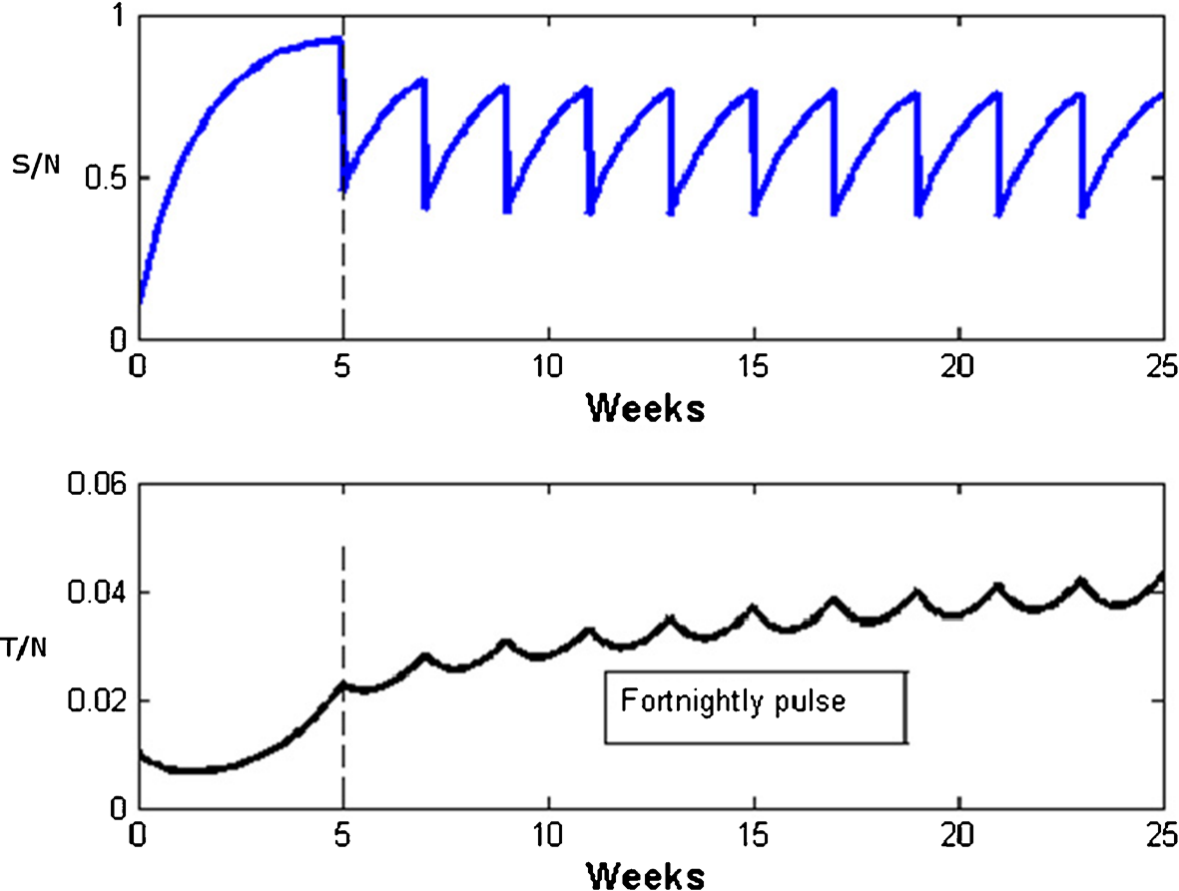
Pulsed STRS model simulations using Eq. (9). The fortnightly impulses abruptly reduce susceptible numbers by *p* = 0.5. Nevertheless, transgressors persist in time. Parameters: α = 0.63; β = 1.5; γ = 0.9; τ = 2; S_0_ = 0.1; T_0_ = 0.01; *p* = 0.5

**Fig. 5:**
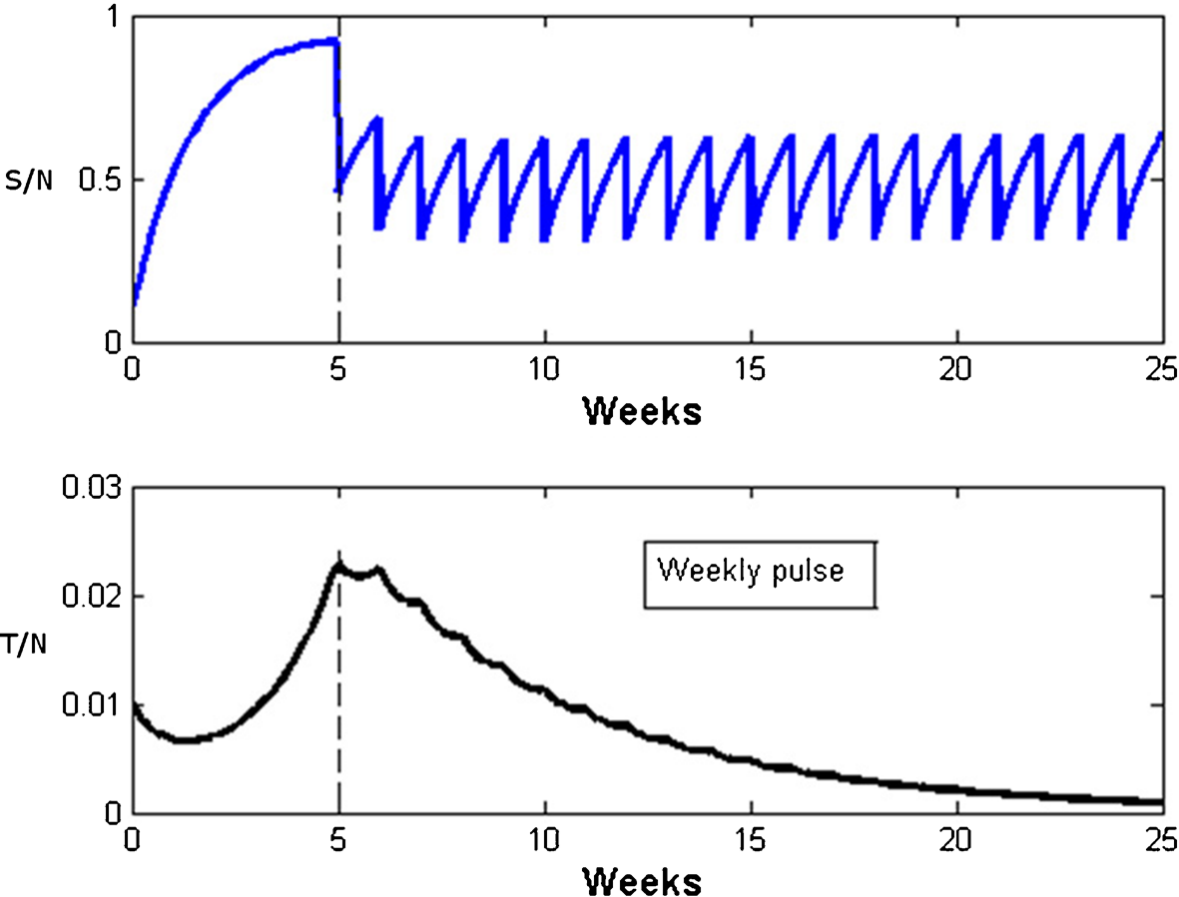
Pulsed STRS model simulations using Eq. (9). The weekly Sabbath impulses abruptly reduce susceptible numbers by *p* = 0.5. Transgressors eventually die out. Parameters: *α* = 0.63; *β* = 1.5; *γ* = 0.9; τ = 1; *S*_0_ = 0.1; *T*_0_ = .01; *p* = 0.5

Figures 4 and 5 show simulations of this model for different values of τ and with *p* = 0.5, so that 50% of susceptible become righteous due to the Sabbath congregation. Figure 4 features a fortnightly Sabbath (τ = 2 weeks) and illustrates how the susceptible numbers suddenly drop by 50% each such Sabbath. Under these conditions, the direct route to righteousness is unable to hold down transgression, which appears to persist permanently in the congregation. In contrast, Fig. 5 shows that with a more frequent weekly Sabbath, transgressors eventually disappear. This is despite the fact that the Basic Sabbath Number is less than unity where transgression should spread unhampered.

A detailed investigation of pulsed equations similar to Eq. (9) may be found in [48, 49]. There, mathematical criteria are derived for determining the threshold point, though these criteria largely fall outside the scope of this paper. In brief though, it is found that the transgressor-free state is stabilized as long as the Basic Sabbath Number ש_0_ is greater than the proportion of susceptibles averaged over τ weeks. The criterion is essentially the same as that for models 1 and 3, except that for the latter models the proportion of susceptibles is unity at the transgression-free steady state. This means the threshold point for Eq. (9) differs by a factor corresponding to the inverse of the average proportion of susceptibles.

**Fig. 6:**
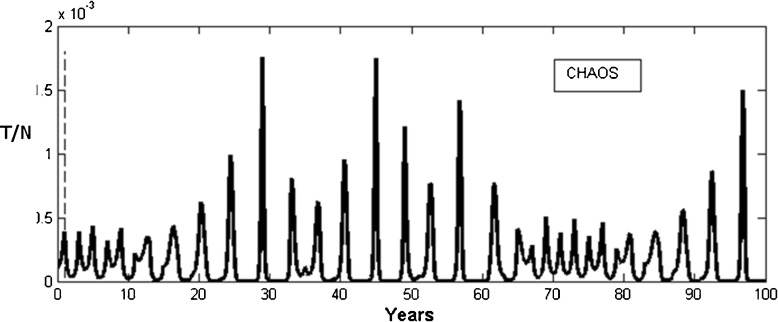
Pulsed STRS model simulations using Eqns. 9 in the chaotic regime. An impulse is given every two years (rather than every week in Figure 5) and abruptly reduce susceptible numbers by *p*=0.5. Transgressors eventually die out. Parameters: α = .00027; β = 10; γ = 1;  τ= 104 weeks; S_0_ = .1; T_0_ = .0001;­ *p* = 0.1

Finally, we note that under some conditions, the pulsed equations (Eq. 9) have the ability to generate complex nonlinear oscillations whereby transgressors exhibit wild chaotic and unpredictable cycles. Figure 6 illustrates one such example whereby in addition to the strong Sabbath repentance (*γ* = 1), a biannual event reduces *S* and increases *R* every 2 years (104 weeks). Although this is admittedly an unusual situation, it demonstrates that periodic annual events can create havoc to the system given the rarity of their occurrence.

## Discussion

### SIR models with periodic coefficients

It was found that in some applications (such as the study of thresholds), the behavior of SIRS models with a periodic coefficient *γ*(t) may be understood from a study of the reduced simpler models having a constant $\gamma =\overset{\_}{\gamma }$ (see Appendix). A similar observation was noted for SIR models with periodic transmission terms *β*(t) [44, 50, 51].

### The age of the Sabbath

Our knowledge about Sabbath assemblies before the Second Temple is based on the biblical account only (Supplementary Information SI1). However, one knows with certainty that Second Temple Israel (516 BCE to 70 CE) observed the Sabbath (see Supplementary Information SI1 [52, 53]). Consequently, the Sabbath institution is at least 2,000 years old. Israel managed its justice administration quasi-autonomously at the end of this period. Section 4 explained the potential stabilizing role of the Sabbath institution in the covenantal societies of Table 1. Rabbinical Judaism is one of these, and it appeared during the Second Temple period.

Pre-monarchic Israel resembled Second Temple Israel: they were both settled, egalitarian societies and surrounded by strong stratified cultures. The archaeological evidence shows that a practically transgression-free society existed in pre-monarchic Israel as far as idolatry [54], pork consumption [13, 54], circumcision [13], and rules prohibiting imported pottery [13] are concerned. The model predicts that if the Sabbath existed in pre-monarchic Israel, then a transgression-free equilibrium was achievable. The mathematical model alone cannot prove a pre-monarchic origin of the Sabbath but it does support it.

### The seventh day: feasibility of a weekly Sabbath and comparison with a bi-weekly assembly

The results of the analysis show that the main stabilizing role of the Sabbath consists in keeping the congregation away from transgression and that requires a favorable above-threshold Basic Sabbath Number (ש_0_ = *γ*/*β* > 1). This can be achieved either by a relatively small *β* or large *γ*. Is a weekly Sabbath sufficient to satisfy ש_0_ = *γ*/*β* > 1? Since *γ* < 1/*τ*, (see S3.4) the numerical answer is that only for *β* ≤ 1 could a weekly Sabbath (*τ* = 1) ensure a transgression-free society. The previous section noted that archaeological excavations revealed negligible idolatry, strict observance of dietary rules, and strict observation of pottery practices. These may be acting together to reduce *β* below unity.

In order to obtain a comparative estimate for the parameter *β*, we take up the metaphor of a transgression that spreads as heroin addiction in modern society. This allows us to use results of Mackintosh and Stewart [46], which imply a contagion rate of *β* = 1.5 (see Model parameters in Section 3 above). Studying this contagion rate can shed light on the hypothetical question as to whether a covenantal society could fight against drug addiction. We thus see that a covenantal society can neither terminate drug addiction spreading with a rate of *β* = 1.5 nor assure a transgression-free society for transgressions spreading with such a rate. This hints that the heroin addiction rate obtained from [46] is faster than the propagation rate of the above-mentioned transgressions of pre-monarchic Israel.

We have seen that a general understanding of the Sabbath model may be gained by choosing the repentance rate *γ* as a constant since some aspects of the model’s behavior are qualitatively similar and sometimes equivalent to the case where *γ* is periodic. Recall that $\overset{\_}{\gamma }$ is directly proportional to the assembly frequency, (see Eq. S3.4) and, assuming reasonably that *α* and *β* remain constant, this allows us to compare weekly and bi-weekly assembly communities. Clearly a weekly repentance rate will be twice as great in a congregation holding a weekly Sabbath than in a congregation assembling bi-weekly, all other things being equal. From this perspective:

- For a given *β*, the Basic Sabbath Number ש_0_ associated with a weekly Sabbath is double that of a bi-weekly Sabbath.
- Consider the proportion of transgressors *T* ^*^/*N* in a congregation that is below the Sabbath threshold. It is easy to see that the transgressors for a weekly Sabbath will be less than half that of the community holding its assemblies once in a fortnight.

These aspects provide some understanding as to why a weekly Sabbath is better able to preserve communities and it outlines the stabilizing role of the Sabbath in covenantal organizations.

Perhaps the most significant question that needs to be addressed is: “Why 7 days?” First we must ask whether a daily Sabbath is feasible. Obviously a daily general assembly would produce a considerable opportunity to win over transgressors, so that the weekly percentage of newly recruited righteous would be impressive. Yet, a society with a daily call for a general assembly is on its way to oligarchy, and should be seen as completely impractical. One might expect that a feasible periodic assembly should be based on the lunar calendar interval of a month. Thus, the next interval would be a natural period as half a month or a month. We now consider reasons why this did not happen.

Ample and consistent evidence collected by other investigations about later communities [55–57] reveals that the chances of a congregation to survive were significantly lower than the chances to fail. This is also consistent with the fact that communities did slip below the threshold. Other communities may have prevailed because they experienced a favorable *β* in the sense that a weekly Sabbath managed to hold them close to the threshold and above it. When taken together, this collective information would imply that in early covenantal communities maintaining a weekly Sabbath, the Basic Sabbath Number was critically close to unity. Changing the Sabbath to a bi-weekly event could reduce the Basic Sabbath Number to well below unity and would also result in a much larger coherence factor. Thus, the weekly Sabbath appears to be the most workable solution. We note, however, that our work found nothing special about the precise interval of 7 days.

Our thesis is that the Fourth Commandment is a concept that covenetal societies latched onto in pre-monarchic Israel from the very beginning of the settlement. This work demonstrates that a weekly Sabbath can control social deviation in covenantal organizations. Consequently, short of an alternative stabilizing mechanism, the Sabbath had to be in place in pre-monarchic Israel when it was most badly needed.

## Conclusions

We note that the model lacks concrete testable predictions, being difficult to validate against archaeological or other evidence. It nevertheless makes a number of interesting predictions:

1. At the conceptual level, the weekly Sabbath was likely viewed as an essential stabilizing function in later covenantal societies.
2. Although no period of assemblies can guarantee with certainty the social stability of a covenantal society, a weekly period appears reasonable.
3. The following covenantal institutions are vital for the survival of a covenantal society:
  Weekly assemblies of the congregation; the strict observance of the Sabbath rules assured compliance with this condition in all the covenantal societies known so far.
  Understanding the concepts of commandment and transgression.
4. Covenantal societies are not easy to copy and even more difficult to invent. They require a proper combination of combating poverty, resisting external influence, avoiding temptation, encouraging righteousness, and observing the Sabbath.
5. This investigation illustrates how mathematical techniques that study epidemics may be useful to understand a specific population equilibrium and stability problem in other contexts. The rich knowledge about SIR models offers quick results and easier investigations of other possible population dynamics applications.

## Electronic supplementary material

Below is the link to the electronic supplementary material.

ESM 1 (DOCX 179 kb)

## Appendix

Threshold of an SIRS with periodic repentance coefficient

We show that the threshold the infection-free steady-state of an SIRS model with periodic *γ*(t), as in Eq. (7), is the same as that found by replacing the function *γ*(t) in Eq. (8) with its mean value $\overset{\_}{\gamma }$. The argument is adapted from [42], which deals with a periodic transmission term. First, recall that a stable transgressor-free equilibrium of model (Eq. 8) is assured if ש_0_ > 1, (see Section 4, above). We first linearize system (Eq. 7) about its transgression-free steady-state to obtain:

$$ \overset{.}{T}=\left[\beta -\gamma (t)\right]T $$

This linear equation is solvable:

$$ T(t)=T(0){e}^{{\displaystyle \underset{0}{\overset{t}{\int }}\left[\beta -\gamma \left(\tau \right)d\tau \right]}}=T(0){e}^{\beta t-{\displaystyle \underset{0}{\overset{t}{\int }}\gamma \left(\tau \right)d\tau }}=T(0){e}^{\left(\beta -\overset{\_}{\gamma}\right)t} $$

Thus, for the above linear system *T*(∞) = 0 *T*(∞) = 0 if ש_0_ = $\frac{\overset{\_}{\gamma }}{\beta }>1$;*T* (∞) → ∞ if *ש*_0_ < 1.

Thus the steady-state of Eqn.7 is stable only if ש_0_ > 1. Yet this is the same criteria as Eq. (8), the SIRS model obtained by replacing the function *γ*(t) in Eq. (7) with its mean value $\overline{\gamma}$.
