# Supplementary material for: The stabilizing role of the Sabbath in pre-monarchic Israel:a mathematical model
Source: J Biol Phys. 2015 Jan 18;41(2):203–21. doi: 10.1007/s10867-014-9373-9 (PMC4366438; doi:10.1007/s10867-014-9373-9)
Supplement: Supplementary file 1 — (DOCX 179 kb) [file 10867_2014_9373_MOESM1_ESM.docx]

|  | **Supplementary Information SI** 1  Testimonies supporting the hypothesis that the Sabbath played an important role of controlling social deviation of covenantal society:   \| Source \| Description \| \| --- \| --- \| \| Old  Testament \| The Bible itself hints that the Sabbath had been kept in Biblical Israel (for example Isaiah 1-13]. The verses demonstrate not only that the established Sabbath preceded Isaiah’s writing, but also that it had enough time to be abused by *transgressors* who sinned behind the façade of Sabbath keepers. Williamson, [S-1]. debates the difficulty of the text. He explains why the “*antiquity of the observance is not in doubt.*”.  Although one is not able to put a date to the text of Isaiah, one can extract from it a testimony that already in biblical times Israelites “religiously” kept the Sabbath. \| \| Research of ancient Israelite  faith \| Counter intuitively, some researchers deduce from the text of the Bible that the Sabbath was not a “*sacred day with worship rituals for ordinary people in the period covered by the Hebrew Bible*”[S-2]. Nevertheless, McKay quotes that it was a day of “*holy rest*” and a day of ”*holy convocation*.” Others argue against McKay’s case [S-3] and maintain that the Sabbath was a day of worship. For the purpose of our research the answer to the question does not matter, because the importance of the Sabbath lies in the assembly rather than the ritual. Our thesis is that in a typical covenantal society the assembly acted as legislator, judge and executioner keeping the congregants on the path of *righteousness*. \| \| Philo of Alexandria \| Philo of Alexandria, [S-4] professes literal observance of the commandments of the Sabbath, because the Sabbath “*maintains the link to those who interpret the traditions literally*.” Philo realizes the importance of the Sabbath assembly, its supreme role of defining the congregation and the values of its congregants. Using the words of Leonhardt-Balzer: “*Every individual who keeps the Sabbath proclaims their adherence to this group; and by attending the Sabbath meetings each member is taught the traditions of the group*.” \| \| Josephus  Flavius \| Josephus [S-5] also testifies that in his time the Sabbath Assembly taught the rules of the congregation and consequently it enabled the Jews to avoid transgression (The source of this conclusion of Barclay is [Ant. 16.43]. \| \| New  Testament \| Similarly, the New Testament reveals to Barclay, [S-5]that the Sabbath “gatherings bound the [Jewish] community together in common loyalty to their distinctive way of life.”. \| \| Cairo  Geniza \| The Cairo Geniza reiterates that the Sabbath assemblies in medieval Egypt kept hosting the forum for "*matters that were in any ways regarded as being of public concern*", [S-6]. \| \| Calvinists  (John  Calvin) \| The primordial role of Sabbath-keeping is a feature not only of Jewish culture but also of other typical covenantal cultures  Most remarkable is John Calvin’s insight into the dependence of the social stability of the congregation and of its social order on the Sabbath assembly. Speaking about the necessity of Sabbath meetings Calvin wrights:  “But unless these meetings are stated and have fixed days allotted to them how can they be held? So impossible, however, would it be to preserve decency and order without this politic arrangement that the dissolution of it would *instantly* lead to disturbance and ruin of the church,” ‎[S-7]. \| \| Scottish  Presbyterians  (Robert  Cox ) \| Robert Cox, [S-8]scrutinizes prisoners of Newgate and subsequently lectures that the path to vice starts with “*evil associates*” and with “*being drawn out by bad associates to the breach of Sabbath.*” The author admits that some Sabbath keepers also stray away, but he explains that nine tenth of the inmates were persons who “were not in the habit of attending a place of worship.” This observation has both a factual value and also an acknowledgment that in the covenantal experience, the mere attendance at the assembly has a significant effect of keeping one on track. \|   B) Since we already blundered into the terminology of political science we first distinguish between egalitarian and stratified societies. In egalitarian societies the members consider each other equal in social status, rights and obligations. There are many variants of egalitarian societies and most of them belong to nomadic cultures: hunter-gatherers, horticulturalists and pastoralists. In settled societies, egalitarian societies are very rare; one form of settled egalitarian culture is the covenantal society which is the focus of this investigation. In contrast, a culture admitting social distinctions between various classes, some more privileged than others is not egalitarian but stratified. Intuitively one assumes that democracy also has its roots in an egalitarian culture, and there is archaeological evidence of pre-bronze age egalitarian settlements in Greece[S-9]. Surprisingly though, the earliest known appearance of democratic rule is in a stratified society ‎[S-9]. Thus, in the democracy of ancient Athens all free men had voting rights (slaves and women had no right to vote). However, not everyone had the right to be elected for every public office. Each function was assigned to a certain social class, which contradicts egalitarian principles even though it is consistent with democracy. We make these distinctions because democracy and the Pilgrims’ covenantal persuasion are fundamental values of modern America. These American values gave rise to the American constitutional confederation, which in turn has been followed all over the world establishing constitutional republics, constitutional monarchies or other constitutional confederations  C) In a covenantal society sovereignty belongs to the congregation. The congregation has the authority to accuse, judge, punish and acquit *transgressors*. In all so-documented covenantal societies (Table 1), members are prohibited to work on the Sabbath and consequently their participation at the weekly assembly is taken for granted. The periodic assembly produced a periodic reinforcement of the group consciousness, of the reliance on each other, of the covenantal obligation resulting in weekly peaks of the social cohesion deterring delinquents from repeating their offenses.    Supplementary Information SI2  This supplement explains the derivation of the eigenvalues of the two equilibria of the STRS model. It also provides additional information about the magnitude of the transgression rate β.  SI2-1 Equilibria and stability  E1: The transgressor-free equilibrium: The Jacobian at the point of equilibrium is $J^{*}=\left[\begin{matrix} -\alpha& (-\alpha-\beta) \\ 0 & \left(\beta-\gamma\right) \end{matrix}\right]$   The eigenvalues need to satisfy the quadratic equation: $\left(-\alpha-\lambda\right)(\beta-\gamma-\lambda)=0$. They are $\lambda_{1}=-\alpha$ and $\lambda_{2}=\beta-\gamma$, and are both real.  When γ>β both eigenvalues of the *transgressor* free equilibrium are negative and the equilibrium is stable. When γ<β, then the eigenvalue λ_2_ is positive and the equilibrium is unstable  E2: The equilibrium of endemic sin The Jacobian is: $J^{*}=\left[\begin{matrix} -\alpha-\frac{\beta\left(1-\frac{\gamma}{\beta}\right)}{1+\frac{\gamma}{\alpha}} & -\left(\alpha+\gamma\right) \\ \frac{\beta\left(1-\frac{\gamma}{\beta}\right)}{1+\frac{\gamma}{\alpha}} & 0 \end{matrix}\right]$   The characteristic equation of the Jacobian is: $\lambda^{2}+\alpha\frac{\alpha+\beta}{\alpha+\gamma}\lambda+\alpha(\beta-\gamma)=0$.  If γ<β then the product of the eigenvalues is positive and their sum is negative, hence both eigenvalues always have negative real parts and the endemic sin equilibrium is stable.  SI2-2 Estimating the rate of spread of heroin addiction   \| **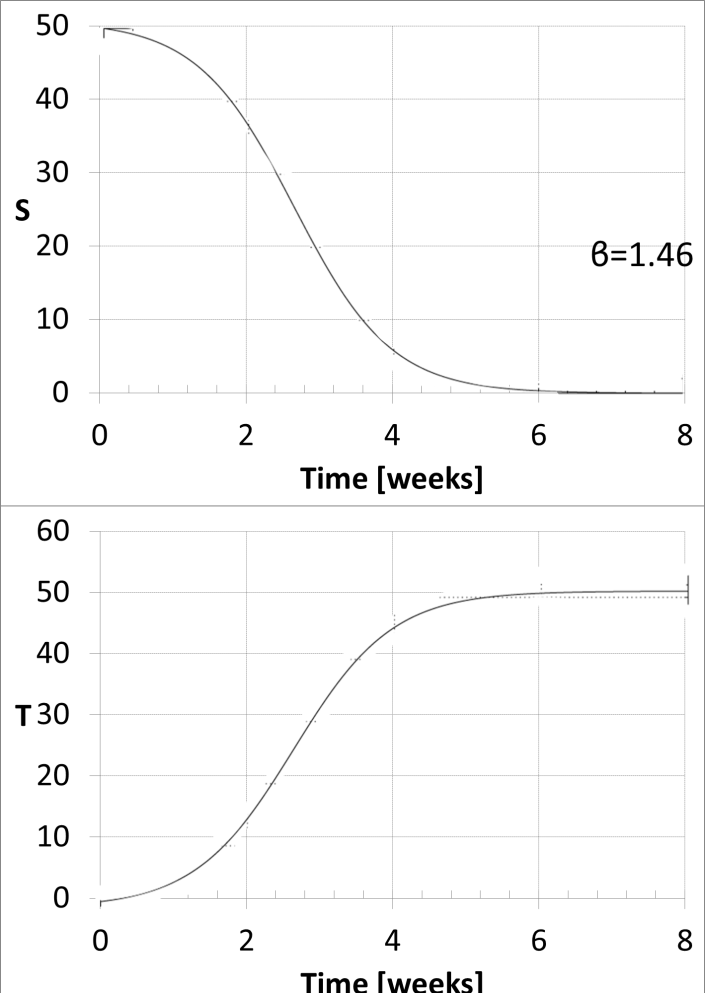** \| \| --- \| \| Figure S2 - 1 Estimating the rate of spread of heroin addiction \|   Figure S2- 1 illustrates the outcome of a simulation replicating results of Mackintosh and Stewart (see Evaluating β of heroin addiction discussed in the main text).  **Supplementary Information SI3**  **SI3-1 The *repentance* coefficient γ**  The section entitled “Model Parameters” of the main article interpreted 1/γ as the mean time to *repentance* (MTTR). This is equivalent to observing that in a population of *T* *transgressors* and with *β*=0 the *transgressors* population will decline at rate of:   \| $\frac{dT}{dt} = -\gamma T$ \| (S3.1) \| \| --- \| --- \|   This has the solution: $T=T_{0}e^{-\gamma t}$ and one can identify 1/γ as the average duration a congregant would keep his/her *transgressor* status, or in other words the mean time to *righteousness* through *repentance* (MTTR). ‎  We now examine the case where γ is a periodic function as represented by its Fourier transform:.   \| $\gamma=\gamma^{\_} +\sum_{n=1}^{\infty} \gamma_{n}\sin(2n\pi t)$ \| (S3.2) \| \| --- \| --- \|   Inserting β=0 and using Equation S3.2 one obtains:   \| $\frac{dT}{dt}=-\gamma T=-T\left[ \bar{\gamma}+\sum_{m=1}^{\infty} \gamma_{n}sin\left( 2n\pi t \right) \right]$ \| (S3.3) \| \| --- \| --- \|   The time average of the harmonic components is zero, [54]. As a consequence, the interpretation of (S3.3) is also valid for a periodic γ or  .  In other words quantifying γ amounts to following a population of *transgressors* T_0_ at time t=0 (marked by the end of the Sabbath) and evaluating their mean time to *repentance*. An MTTR shorter than the duration of a period means that at least one *transgressor* has repented before the first Sabbath after t=0. An MTTR shorter than one period therefore implies a society in which *repentance* is not caused only by the Sabbath assembly but also by additional factors contradicting our hypothesis. Therefore in our investigation the domain of interest must be:   \| $MTTR=\frac{1}{\bar{\gamma}}\geq\tau$ \| or \| $\bar{\gamma}\leq\frac{1}{\tau}$ \| (S3.4) \| \| --- \| --- \| --- \| --- \|   SI3-2 Responses S, T  This section examines the average responses: $\overline{S},\overline{T}$and $\overline{R}$ of an SIRS model (Eqn.7) with a periodic γ(*t*) at steady state. The section shows that these responses will settle at values that are very close to the corresponding values given by (4), the transgression free equilibrium of SIRS model eqn.8, for which γ(t) is replaced by the constant $\overline{\gamma}$.  Fourier series of responses  The periodic nature of the covenantal lifestyle suggests, and Theorem 5 of [S-10] proves, that the solution of an STRS system is also of the form:   \| $S\left( t \right)=\bar{S}+\sum_{n=1}^{\infty} S_{n}\sin\left( 2\pi nt+\Phi_{Sn} \right)$  $T\left( t \right)=\bar{T}+\sum_{n=1}^{\infty} T_{n}\cos\left( 2\pi nt+\Phi_{Tn} \right)$  $R\left( t \right)=N-S\left( t \right)-T(t)$ \| (S3.5) \| \| --- \| --- \|   *S_n_, T_n,_ Φ_Sn_, and Φ_Tn_* of (S3.5) are harmonic amplitudes and phase angles determined according to Fourier series formulae (see for example page 197 of [A6]).  Zero Frequency Terms  Inserting (S3.5) into Eq. (3) and separating the zero frequency terms one obtains:   \| $\begin{matrix} 0=\alpha\left(N-\mathop{S}\limits^{\_} -\mathop{T}\limits^{\_} \right)-\frac{\beta S^{*}\mathop{T}\limits^{\_} }{N}-\frac{\beta}{N}\frac{1}{2}\sum_{n=1}^{\infty} S_{n}T_{n}\sin\left(\varphi_{Sn}+\varphi_{Tn}\right) \\ 0=\frac{\beta\mathop{S}\limits^{\_} \mathop{T}\limits^{\_} }{N}+\frac{\beta}{N}\frac{1}{2}\sum_{n=1}^{\infty} S_{n}T_{n}\sin\left(\varphi_{Sn}+\varphi_{Tn}\right)-\gamma^{\_} \mathop{T}\limits^{\_} -\frac{1}{2}\sum_{n=1}^{\infty} \gamma_{n}T_{n}\sin\varphi_{Tn} \end{matrix}$ \| (S3.6) \| \| --- \| --- \|   The *zero frequency terms* are the averages of the responses at steady state.  When at steady state The second row of (3) is: $\begin{matrix} \mathop{\left(\frac{dT}{T}\right)}\limits=\beta S(t)/N-\gamma(t) \\ \end{matrix}$     The interpretation of this form is that the growth rate of *transgressors* is an algebraic sum of two opposed variables: the increment due to corruption and the reduction due to *repentance*. But when steady state is reached the average growth rate of *transgressors* becomes zero. Therefore the right hand side must also average to zero:   \| $\int_{0}^{\tau} \left[ \beta\frac{S(t)}{N}-\gamma(t) \right]dt=0$ \| or: \| $\frac{\beta\bar{S}}{N}=\bar{\gamma}$ \| \| --- \| --- \| --- \|   This finally leads to:   \| $\overline{S}=S^{*}$ \| (S3.7) \| \| --- \| --- \|   As a consequence the first row of (S3.5) can be written also as:    Since $\frac{\beta S^{*}\bar{T}}{N}-\bar{\gamma}\bar{T}=0$ the second row of (S3.6) becomes:   \| $\frac{\beta}{N}\frac{1}{2}\sum_{n=1}^{\infty} S_{n}T_{n}\sin\left(\varphi_{Sn}+\varphi_{Tn}\right)=\frac{1}{2}\sum_{n=1}^{\infty} \gamma_{n}T_{n}\sin\varphi_{Tn}$ \| (S3.8) \| \| --- \| --- \|   Inserting (S3.8) into the first row of (S3.6) and defining, one obtains:   \| $-\left(\alpha+\frac{\beta S^{*}}{N}\right)\Delta T=\frac{\beta}{N}\frac{1}{2}\sum_{n=1}^{\infty} S_{n}T_{n}\sin\left(\varphi_{Sn}+\varphi_{Tn}\right)$ \| (S3.9) \| \| --- \| --- \|   Neglecting the higher harmonics one can show that:**.**   \| $\left\vert\frac{\Delta T}{N}\right\vert=\frac{\frac{\gamma_{1}}{2}\frac{T_{1}}{N}\sin\varphi_{T1}}{\alpha+\gamma^{\_} }=\frac{\gamma_{1}}{\mathop{2\gamma}\limits^{\_} }\frac{T_{1}}{N}\frac{\sin\varphi{}_{T1}}{\left(1+\alpha/\gamma^{\_} \right)}$ \| (S3.10) \| \| --- \| --- \|   Table S3-1compiles simulation results reached at steady state of a group of N=100 people for ש_0_ varying from high to low within the endemic sin domain. The cohesion factor of the simulations of Table S3-1 is 1. The simulation results verify Eq. (S3.7). The last column shows that all along the endemic sin domain ΔT/N is very small.  The excursions of the periodic terms S_1_ and T_1_ are small the largest being T_1_/N=8.5%. This justifies neglecting higher harmonics for the derivation of the relationships of (S3.9). The low values for T_1_ and φ_T1_ explain why ΔT/N is small for the cohesion factor of 1. (S3.10) predicts that when the cohesion factor approaches zero ΔT/N also approaches zero. Since $\left( 1+\alpha/\bar{\gamma} \right)\geq1$(S3.10) guarantees that the increment \|ΔT/N\| cannot exceed the double of the values obtained in Table S3-1. As a result for practical purposes $\bar{T}$is the same as $T^{*}$.  Table ‎S3‑1Simulation Results; N=100; Cohesion Factor=1   \| ש_0_ \| S* \| T* \| $\bar{\boldsymbol{S}}$ \| $\bar{\boldsymbol{T}}$ \| S_1_ \| T_1_ \| φ_T1_ [rad] \| ΔT/N - simulation \| \| --- \| --- \| --- \| --- \| --- \| --- \| --- \| --- \| --- \| \| 0.800 \| 80.000 \| 10.000 \| 80.002 \| 10.001 \| 0.50 \| 1.60 \| 0.07 \| 5.00E-06 \| \| 0.667 \| 66.667 \| 16.667 \| 66.667 \| 16.670 \| 0.84 \| 2.68 \| 0.07 \| 3.03E-05 \| \| 0.571 \| 57.143 \| 21.429 \| 57.143 \| 21.434 \| 1.08 \| 3.47 \| 0.06 \| 5.33E-05 \| \| 0.500 \| 50.000 \| 25.000 \| 50.000 \| 25.008 \| 1.26 \| 4.07 \| 0.07 \| 8.10E-05 \| \| 0.333 \| 33.333 \| 33.333 \| 33.333 \| 33.357 \| 1.68 \| 5.55 \| 0.07 \| 2.41E-04 \| \| 0.250 \| 25.000 \| 37.500 \| 25.000 \| 37.548 \| 1.88 \| 6.38 \| 0.08 \| 4.77E-04 \| \| 0.200 \| 20.000 \| 40.000 \| 20.000 \| 40.078 \| 1.99 \| 6.93 \| 0.10 \| 7.77E-04 \| \| 0.050 \| 5.000 \| 47.500 \| 4.999 \| 48.002 \| 1.37 \| 8.47 \| 0.32 \| 5.02E-03 \| \| 0.020 \| 2.000 \| 49.000 \| 2.000 \| 49.601 \| 0.61 \| 8.09 \| 0.35 \| 6.00E-03 \|   Conclusions   1. The STRS model forced by a periodically fluctuating γ converges to a steady state. 2. The steady state responses consist of a constant average plus a time history that repeats itself every period. 3. The average of the steady state S-response is identical to S*. The average of the T-response is not identical but for practical purposes it is equal to T*. 4. The average of the *repentance* coefficient is limited or:$\bar{\gamma}$τ≤1. 5. When the Basic Sabbath Number is above the threshold the periodically forced model behaves exactly as the zero frequency unforced model and it converges to a transgression-free equilibrium. In this range the two models are interchangeable. 6. When the Basic Sabbath Number slips a little below the threshold the periodic contributions to the steady state responses are negligible. 7. Even when the Basic Sabbath Number is below the threshold the periodic responses are of minor importance and the zero frequency unforced model provides effective insight. |
| --- | --- | --- | --- | --- | --- | --- | --- | --- | --- | --- | --- | --- | --- | --- | --- | --- | --- | --- | --- | --- | --- | --- | --- | --- | --- | --- | --- | --- | --- | --- | --- | --- | --- | --- | --- | --- | --- | --- | --- | --- | --- | --- | --- | --- | --- | --- | --- | --- | --- | --- | --- | --- | --- | --- | --- | --- | --- | --- | --- | --- | --- | --- | --- | --- | --- | --- | --- | --- | --- | --- | --- | --- | --- | --- | --- | --- | --- | --- | --- | --- | --- | --- | --- | --- | --- | --- | --- | --- | --- | --- | --- | --- | --- | --- | --- | --- | --- | --- | --- | --- | --- | --- | --- | --- | --- | --- | --- | --- | --- | --- | --- | --- | --- | --- | --- | --- | --- | --- | --- | --- | --- | --- | --- | --- | --- | --- | --- | --- | --- | --- | --- | --- | --- | --- | --- | --- |

References

S-1. Williamson, H.G.M.: A Critical and Exegetical Commentary on Isaiah 1-27: Commentary on Isaiah 1-5. Continuum International Publishing Group, New York (1996)

S-2. McKay, H.A.: Sabbath and Synagogue: the Question of Sabbath Worship in Ancient Judaism. BRILL, Boston (2001)

S-3. Van der Horst, P.W.: Japheth in the tents of Shem: studies on Jewish Hellenism in antiquity. Peeters Publishers, Leuven (2002)

S-4. Leonhardt, J.: Jewish Worship in Philo of Alexandria. Mohr Siebeck, Tübingen (2001)

S-5. Barclay, J.M.G.: Jews in the Mediterranean Diaspora: from Alexander to Trajan (323 BCE - 117 CE). University of California Press, Berkeley, CA (1999)

S-6. Goitein, S.D. and J. Lassner: A Mediterranean Society: An Abridgment in One Volume. University of California Press., Berkley (1999)

S-7. Kingdon, R.M.: John Calvin's Contribution to Representative Government,. In P. Mack and M.C. Jacob, Editors. Politics and Culture in Early Modern Europe, pp. 183-199. Cambridge University Press: Cambridge, U.K. (1987)

S-8. Cox, R.: Sabbath Laws and sabbath Duties. Maclachlan and Stewart, Edinburgh (1853)

S-9. Wilson, N.G.: Encyclopedia of Ancient Greece. Routledge, New York (2006)

S-10. Wylie, J.R. and R. Clarence: Advanced Engineering Mathematics. McGraw Hill, New York (1966)
